# Extracellular Vesicle-Mediated siRNA Delivery, Protein Delivery, and CFTR Complementation in Well-Differentiated Human Airway Epithelial Cells

**DOI:** 10.3390/genes11040351

**Published:** 2020-03-26

**Authors:** Brajesh K. Singh, Ashley L. Cooney, Sateesh Krishnamurthy, Patrick L. Sinn

**Affiliations:** Stead Family Department of Pediatrics, Carver College of Medicine, The University of Iowa, Iowa City, IA 52242, USA; Ashley-peterson@uiowa.edu (A.L.C.); sateesh-krishnamurthy@uiowa.edu (S.K.); patrick-sinn@uiowa.edu (P.L.S.)

**Keywords:** exosomes, microvesicles, cystic fibrosis, lung, primary cells

## Abstract

Extracellular vesicles (EVs) are a class of naturally occurring secreted cellular bodies that are involved in long distance cell-to-cell communication. Proteins, lipids, mRNA, and miRNA can be packaged into these vesicles and released from the cell. This information is then delivered to target cells. Since EVs are naturally adapted molecular messengers, they have emerged as an innovative, inexpensive, and robust method to deliver therapeutic cargo in vitro and in vivo. Well-differentiated primary cultures of human airway epithelial cells (HAE) are refractory to standard transfection techniques. Indeed, common strategies used to overexpress or knockdown gene expression in immortalized cell lines simply have no detectable effect in HAE. Here we use EVs to efficiently deliver siRNA or protein to HAE. Furthermore, EVs can deliver CFTR protein to cystic fibrosis donor cells and functionally correct the Cl^−^ channel defect in vitro. EV-mediated delivery of siRNA or proteins to HAE provides a powerful genetic tool in a model system that closely recapitulates the in vivo airways.

## 1. Introduction

Primary cultures of well-differentiated human airway epithelial cells (HAE) are a robust model for studying epithelial cell biology. Cells grown at an air–liquid interface form a polarized, pseudostratified columnar epithelium that closely resembles the morphology of the in vivo surface epithelium of the conducting airways [1,2,3]. This model provides an opportunity to study cell biology, disease progression, pathogenesis, and treatments for lung diseases like cystic fibrosis [4]. However, well-differentiated HAE are refractory to transfection techniques for delivering expression plasmids, small interfering RNA molecules (siRNA) [5,6,7,8], and single-stranded oligonucleotides [5,6]. Transfection is possible at the time of seeding when the cells are still poorly differentiated [9]. Transfecting siRNA into poorly differentiated airway cells leads to knockdown of target genes; however, this strategy has its limitations because poorly differentiated HAE are simply less representative of the in vivo airways. As a result, viral-based vectors (such as adenovirus, lentivirus, or adeno-associated virus) are typically employed to deliver genetic material to HAE. Viral vectors can be expensive and time consuming to generate. To date, many effector molecules and approaches have been investigated to increase the transfection efficiency in HAE [7,9]; yet despite much effort, there remains a need to identify an efficient delivery strategy in this in vitro model system. The ability to efficiently transfect well-differentiated HAE would open the doors to wide-ranging experimental questions that are currently not feasible, such as probing novel gene targets for therapeutic rescue of cells lacking cystic fibrosis transmembrane conductance regulator (CFTR) function [9,10,11,12].

Extracellular vesicles (EVs) are a family of lipid-bound cellular bodies that are secreted by most cells in the body, including T cells, B cells, dendritic cells, mast cells, and epithelial cells [13,14,15,16]. EVs can be purified from several types of extracellular body fluids including blood, urine, amniotic fluid, breast milk, saliva, and cerebrospinal fluid [17]. EVs are also readily collected from the supernatant of various cultured cell types in vitro [18]. The two main types of EVs are microvesicles (MVs) and exosomes, which are classified based upon their biogenesis, release pathways, size, content, and function [19,20]. These vesicles were once thought to be a mechanism for removing unwanted proteins, but many studies show that EVs are involved in intercellular communications. For example, they package highly variable cargo and facilitate transfer of proteins, lipids, microRNAs, and mRNAs from donor cells to target cells [21,22,23,24,25,26]. Exosomes bind a wide range of surface receptors (tetraspanins, integrins, CD11b and CD18 receptors) and are internalized by phagocytosis or endocytosis [27]. MVs endocytose or potentially fuse to the cell surface; thereby, integrating lipids and proteins directly into the plasma membrane of target cells [19]. Both MVs and exosomes are involved in many cellular processes and evolved to deliver multiple types of genetic and other cargo to a wide range of cellular targets [27,28,29,30,31].

Here we demonstrate the utility and functional impact of exosome-mediated delivery of siRNA and MV-mediated delivery of CFTR protein in well-differentiated HAE. Our data provide evidence that EVs can provide a rapid, inexpensive, and robust tool to deliver small RNAs and proteins into an important model system.

## 2. Materials and Methods

### 2.1. Cell Culture

Primary cultures of human airway epithelia were cultured from trachea and bronchi as described previously [2]. Briefly, epithelial cells suspensions were enzymatically dissociated and seeded at a density of 5 × 10^5^ cells/cm^2^ onto collagen-coated, Transwell inserts (Corning Costar polycarbonate filters) with 0.6 cm^2^ semipermeable support membranes. The cells were then incubated for 24 h at 37 °C and 5% CO_2_. The apical medium was removed and the cells were maintained in Ultroser G (Biosepra SA, Cedex, France) medium for more than 4 weeks at an air-liquid interface. All studies described in this article received Institutional Review Board approval (IRB ID 9507432).

### 2.2. Isolation of Extracellular Vesicles (EVs)

**Figure 1:**
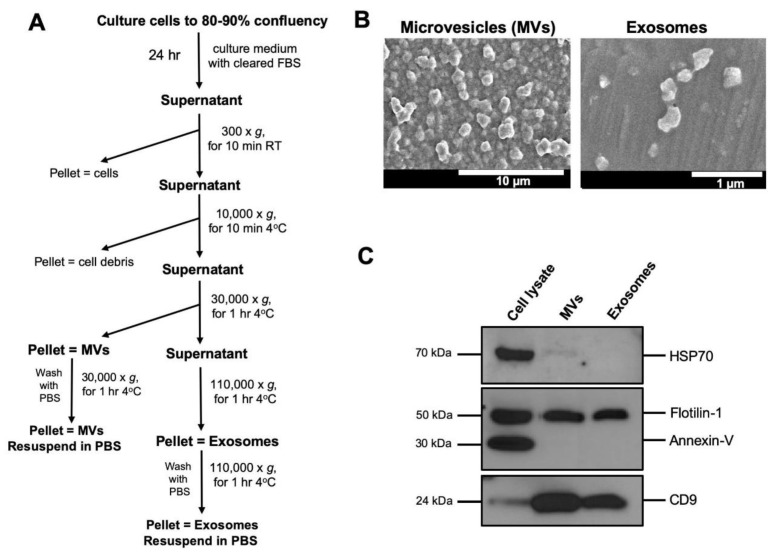
Isolation and characterization of extracellular vesicles (EVs) from A549 cells. (**A**) The workflow of differential ultracentrifugation for extracellular vesicles isolation is shown. (**B**) Scanning electron microscopy of microvesicles (MVs) (left panel) (scale bar = 10 µm) and exosomes (right panel). Scale bar = 1 µm. EVs were isolated from A549 cells cell culture medium by differential ultracentrifugation at 30,000× *g* and 110,000× *g*, respectively, and negatively stained for observation under the EM. (**C**) Proteins from whole cell lysates, MVs, and exosomes from cultured cells were separated on SDS-PAGE, followed by Western blotting using antibodies against CD9, HSP70, Flotilin-1, and Annexin V.

To avoid contamination with fetal bovine serum (FBS) derived EVs, FBS was centrifuged at 110,000× *g* for 2 h and the pellet was discarded. The cell culture media of HEK-293T or A549 cells was replaced with DMEM supplemented with EV cleared FBS one day before supernatant collection. The cell culture supernatants were collected and the EVs were isolated by differential centrifugation as previously described [18,32]. EVs are typically isolated from 60–80 mL of culture supernatant. We collect this supernatant from ~1.5 × 10^8^ cells growing at confluency from 4 × 150 mm culture dishes. Briefly, the cell supernatants were cleared of intact cells and cell debris by centrifugation at 300× *g* for 10 min and 10,000× *g* for 10 min at 4 °C, before the first ultracentrifugation at 30,000× *g* for 70 min at 4 °C to pellet MVs (Figure 1A). The supernatant was then filtered through a 0.22 µm Amicon filter before the second round of ultracentrifugation at 110,000× *g* for 70 min at 4 °C to pellet exosomes. The MV and exosome pellets were washed in phosphate buffered saline (PBS) once and re-suspended in 200 µl of PBS or culture media with gentle mixing and stored at −80 °C. The recovery of EVs was estimated by measuring the protein concentration using a Bradford assay. The protein concentrations varied from 0.25 to 0.5 µg/µL.

### 2.3. Electron Microscopy

Isolated EVs were fixed with 2.5% glutaraldehyde in 0.1 M sodium cacodylate buffer for 2 h at room temperature. Fixed exosomes suspensions were deposited on formvar/carbon-coated grids (Ted Pella Inc, Redding, CA, USA) for 20 min. Grids were then washed with two rinses of buffer and post-fixed with 1% osmium tetroxide for 1 h. The grids were dehydrated through ascending grades of ethanol (25%, 50%, 75%, 95% and 100% ethanol). Grids were rinsed two times with hexamethyldisilazane (HMDS) for 15 min each and then air dried overnight. The grids were placed on aluminum stubs, sputter coated and visualized under a Hitachi S-4800 scanning electron microscope (Hitachi High Technologies America Inc., Pleasanton, CA, USA).

### 2.4. SDS-PAGE and Immunoblot Analysis

Total protein lysates were prepared from HAE and EVs in freshly prepared lysis buffer (1% Triton, 25 mmol/L Tris pH 7.4, 150 mmol/L NaCl) containing protease inhibitors (complete; mini, EDTA-free; Roche Biochemicals, Mannheim, Germany) for 30 min at 4 °C. Five µg of prepared lysates were solubilized in Laemmli reducing sample buffer (Bio-Rad, Hercules, USA) and separated on a 10% precast Mini-PROTEAN TGX gel (Bio-Rad, Hercules, CA, USA). Proteins were electro transferred onto PVDF membrane (Millipore, Burlington, MA, USA), and the membranes were blocked in 5% (*w*/*v*) skim milk powder in Tris-buffered saline with 0.05% (*v*/*v*) Tween-20 (TBST) for 1 h at RT. Membranes were probed with anti-flotilin-1, anti-CD9, anti-HSP70, and anti-annexin V from the Exosomal Marker Antibody Sampler Kit (Cell Signaling Technology, Danvers, MA, USA) diluted in TBST for 1 h. Blots were rinsed and incubated in horseradish peroxidase-conjugated goat anti-sheep IgG (Bio-Rad, Hercules, MA, USA; 1:10,000) or goat anti-mouse IgG (Bio-Rad, Hercules, MA, USA; 1:10,000) for 1 h at RT with shaking. Antibody staining was visualized by chemiluminescence (ECL Plus Western blotting detection reagents, GE Healthcare, Pittsburgh, PA, USA).

### 2.5. Dicer-Substrate Short Interfering RNA (DsiRNA) Oligonucleotides

The 27-mer DsiRNAs and digoxigenin (DIG)-labeled DsiRNA were designed and synthesized by Integrated DNA Technologies (IDT, Coralville, IA, USA) as described earlier [33,34,35]. The DIG label was internally coupled to an amino-dT base in a 2-O′ methyl modified DsiRNA against hypoxanthine-guanine phosphoribosyltransferase (HPRT). The sequences of DsiRNAs used in the study are provided in Table 1.

### 2.6. Loading of DsiRNA into Exosomes

Exosomes were loaded with DsiRNA or DIG-labelled DsiRNA by electroporation as previously described [36]. Briefly, exosomes and DsiRNA were mixed in a 1:1 ratio (wt/wt) in electroporation buffer to a final concentration of 250 ng/µL. The exosome-DsiRNA mixtures were electroporated in a 400 µL volume using 0.4-mm cuvettes at 400 mV and 125 μF capacitance with pulse time of 10–15 ms. To remove non-electroporated DsiRNA, electroporated exosomes first treated with RNase and then washed in 1.5 mL of PBS, ultracentrifuged at 110,000× *g* for 70 min and re-suspended in 50 μL of culture media.

### 2.7. Exosome Labeling

Exosomes were stained with 1X CellMask Deep Red Plasma Membrane stain (Thermo Fisher Scientific, Waltham, MA, USA) for 10 min at 37 °C. Unbound CellMask stain were then washed by ultracentrifugation at 110,000× *g* for 70 min in TLA 100.3 rotor (Beckman Coulter, Indianapolis, IN, USA). Finally, exosomes were re-suspended in 100 µL of culture media and stored at −80 °C for further analysis. 

### 2.8. Confocal Microscopy and Immunostaining

For confocal microscopy, HAE were fixed in 2% paraformaldehyde, permeabilized with 0.2% Triton-X-100 in Superblock (Thermo Fisher Scientific, Waltham, MA, USA), and then blocked in 1× Superblock for 1 h at RT. F-actin was stained with either rhodamine-phalloidin (1:100, cat. no. R415, Thermo Fisher Scientific, Waltham, MA, USA) or Alexa Fluor 488-phalloidin (1:100, cat. no. A12379, Thermo Fisher Scientific, Waltham, MA, USA) for 30 min at RT. For immunostaining, HAE were incubated with primary antibody against mouse anti-CFTR monoclonal antibody (769, CFFT) or anti-Digoxygenin (cat. no. 11333089001, Roche Biochemicals, Mannheim, Germany) overnight at 4 °C. HAE were then incubated for secondary antibodies was Alexa 488-labeled goat anti-mouse or Alexa 488-labeled goat anti-sheep for 1 h at RT. HAE were mounted on a slide with Vectashield with DAPI (Vector Laboratories Inc., Burlingame, CA, USA). Representative images from three donors are shown. Z-stacks were acquired on a Leica TCS SP3 confocal microscope (Leica Microsystems Inc., Buffalo Grove IL, USA) using a 40× or 63× oil-immersion objective. 

### 2.9. Real-Time Quantitative PCR (RT-qPCR)

Total cellular RNA was isolated using Direct-zol™ RNA MiniPrep kit (Zymo Research, Irvine, CA, USA), according to the manufacturer’s protocol. The RNA concentration of samples was quantified using ND-1000 spectrophotometer (Thermo Fisher Scientific, Waltham, USA). 0.5 µg of total RNA was reverse transcribed by a high-capacity reverse transcription kit (Thermo Fisher Scientific, Waltham, MA, USA) according to the manufacturer’s instructions. qRT-PCR for HPRT and SFRS9 mRNA were performed in an ABI Prism 7900 HT real-time PCR system (Thermo Fisher Scientific, Waltham, MA, USA). The PCR conditions were as follows: 95 °C for 10 min, 95 °C for 15 s, and 60 °C for 1 min for 40 cycles. The following primers were used in RT-qPCR analysis: For hHPRT AGGATTTGGAAAGGGTGTTTATTC (forward) and CCCATCTCCTTCATCACATCTC (reverse) and for hSFRS9: TGCGTAAACTGGATGACACC (forward) and CCTGCTTTGGTATGGAGAGTC (reverse). The mRNA level of HPRT was calculated with normalization to SFRS9 using the 2^−∆∆CT^ method. Fold change in expression are means of three technical triplicates from three human donors.

### 2.10. Isolation of CFTR- or mCherry-Loaded EVs and Delivery to HAE

To isolate EVs loaded with either mCherry or CFTR, A549 cells were transfected with an expression plasmid or transduced with an adenovirus expressing mCherry (Ad5-mCherry) or CFTR (Ad5-CFTR) under the direction of a short 183-bp synthetic enhancer/promoter F5tg83 as previously described [37,38]. Briefly, cells were grown to 70–80% confluency and transduced at MOI = 50 for 4 h. After transduction, cells were washed three times with PBS to wash away unbound viral particles. Fresh culture medium (DMEM supplemented with EV-cleared FBS) was added to the cells. After 48 h EVs were isolated as described above.

To treat the airway epithelia with EVs apically, HAE were first treated apically with 0.1% lysophosphatidylcholine (LPC) in PBS for 2 h. HAE were rinsed three times with PBS before EVs were applied in a volume of 100 µL culture media for 4 h at 37 °C. EVs were removed and HAE were incubated at 37 °C under 5% CO_2_ for the indicated times. In the case of basolateral application, the cultures were inverted, and the EVs were applied to the basolateral surface for 4 h in 80 µl of culture media at 37 °C, 5% CO_2_. Following the treatment, the EVs were removed, and the cultures were turned upright and incubated at 37 °C under 5% CO_2_ for the indicated times.

### 2.11. Ussing Chamber Studies of Well-Differentiated HAE

To measure change in anion channel activity, HAE were mounted into Ussing chambers as described earlier [39]. The apical and basolateral chambers were filled with symmetrical Ringer’s solution (135 mM NaCl, 5 mM HEPES, 0.6 mM KH_2_PO_4_, 2.4 mM K_2_HPO_4_, 1.2 mM MgCl_2_, 1.2 mM CaCl_2_, 5 mM dextrose). Dextrose was added to this solution immediately before the experiments. The protocol was performed as follows: treatment with amiloride (100 µM), followed by DIDS (100 µM), apical solution was replaced with a low Cl^−^ solution, and CFTR Cl^−^ current was measured as previously described [40]. 

## 3. Results

### 3.1. Isolation and Characterization of Extracellular Vesicles

EVs were isolated from culture media of HEK-293T or A549 cells. EVs were fractionated into two populations by differential ultracentrifugation. The first fraction, containing microvesicles (MVs), was recovered from the pellet after centrifugation at 30,000× *g*, and the second fraction, containing exosomes, was recovered from the pellet after centrifugation at 110,000× *g* (Figure 1A). We next characterized the two fractions for their morphology/size and protein surface markers (Figure 1B,C). As expected, the MV fraction consisted of large vesicles ranging from 300–1000 nm in diameter (Figure 1B, left panel), whereas the second exosome fraction contained smaller size vesicles ranging from 80–150 nm (Figure 1B, right panel). To further confirm the purity of our fractions, proteins from cell lysates, MVs, and exosomes were characterized by immunoblot analysis for EVs specific protein markers [41] (Figure 1C). Tetraspanin-like CD9 and flotillin-1 were highly enriched in the EVs, whereas HSP70 and annexin V were not enriched in either the MV or exosome fractions. HSP70 was not detectable in the exosome fraction, but faintly present in the MV fraction, suggesting that large vesicles contain cellular proteins. This suggests the probable differences in biogenesis, where exosomes are secreted through the endosome pathway and would present endosome markers, whereas larger vesicles such as MVs are produced by direct budding from the cell membrane and would contain cellular proteins or proteins associated with the plasma membrane. The absence of annexin V from the EV fractions confirms the absence of apoptotic bodies in our EV preparations. Our results are consistent with previous reports that describe the protein composition in exosomes and MVs [19,28].

### 3.2. Exosomes are Internalized at the Basolateral Surface of HAE

**Figure 2:**
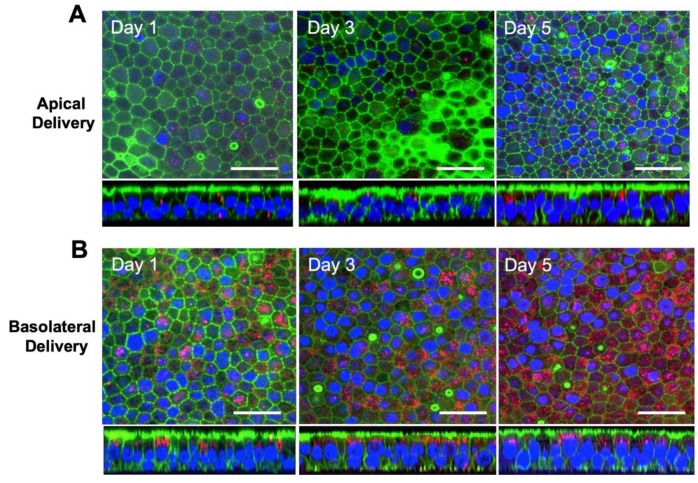
Exosomes can be delivered to well-differentiated HAE. Exosomes (5 µg) were labelled with CellMask Deep Red Plasma Membrane stain and applied apically (**A**) or basolaterally (**B**) to HAE for 4 h. HAE were fixed, permeabilized, and then stained for F-actin (green) and nuclei (blue). HAE were examined by confocal microscopy for internalization of exosomes at the indicated time points. Scale bar = 50 µm.

For our initial studies, we asked whether EVs would be taken up by well-differentiated HAE and whether there is a preference for the apical or basolateral surface. Our standard protocol requires the HAE cultures to be grown on polycarbonate Transwell inserts with a pore size of 400 nm. Because this pore size physically limits the access of MVs to the basolateral surface, we focused our efforts on exosomes which are smaller than the pore size of the Transwell insert. To evaluate the efficiency of exosome uptake by HAE, exosomes derived from HEK-293T cells were labeled with CellMask Deep Red Plasma Membrane stain and applied to the apical (Figure 2A) or basolateral (Figure 2B) surface of HAE for 4 h. At the indicated time points post-delivery, confocal microscopy was used to evaluate intracellular uptake of CellMask stain. Following apical exosome delivery, few (estimated <1%) CellMask positive cells were observed (Figure 2A). In contrast, following basolateral exosome delivery, we readily observed abundant CellMask positive HAE (Figure 2B). Exosome internalization was observed as early as 3 h after basolateral application. Regardless of apical or basolateral exosome delivery, the CellMask stain was stable for at least 5 days after delivery and remained localized in perinuclear regions below the apical surface. However, the presence of CellMask is not an indication of the stability of internalized exosomes, only the internalized labeled lipid bilayers. These data suggest that exosomes are an efficient delivery vehicle to HAE and that delivery is more efficient from the basolateral surface than the apical surface.

### 3.3. Delivery of siRNA into HAE Using Exosomes

**Figure 3:**
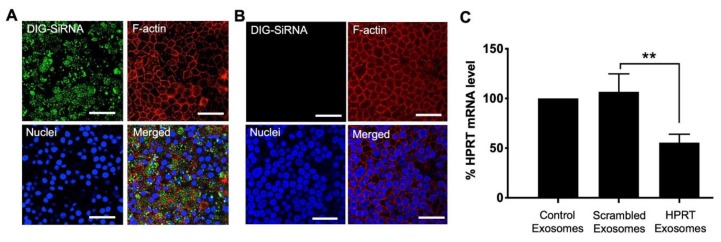
Exosome-mediated delivery of DsiRNA effectively silences target gene in HAE. DsiRNA can be delivered into HAEs using exosomes. HAEs were treated for 4 h with A549 derived exosomes (**A**) electroporated with digoxigenin (DIG) labeled siRNA (green) or (**B**) non-electroporated exosomes. After 24 h, HAE were fixed, permeabilized, and immunostained for DIG-labelled DsiRNA with anti-digoxygenin (green) and for F-actin (red) and nuclei (blue). HAE were examined using confocal microscopy. Scale bar = 50 µm. (**C**) qRT-PCR of HPRT mRNA in exosome treated cells. HAE were treated for 4 h with 10 μg of A549 derived exosomes electroporated with scrambled or siRNA specific to HPRT gene. After 24 h HPRT mRNA levels were measured using qRT-PCR. HPRT mRNA was normalized against reference gene SFRS9 in HAE. All experiments were performed in triplicate from three donors. Each repeat was carried out with a unique exosome preparation. Data are represented as means and s.d. of fold change from 3 donors, ** *p* < 0.01.

To test if EVs could be used to functionally modify gene expression in HAE, we used exosomes to deliver siRNA and quantified the level of knockdown. Because of the clear basolateral preference of exosomes, we focused on basolaterally applied exosomes. Using the previously described protocol (Figure 1A), exosomes were purified from the supernatant of A549 cells. The exosomes were then electroporated in the presence of digoxigenin (DIG)-labeled siRNA. Exosomes containing DIG-siRNA were applied to the basolateral surface of the HAE cultures for 4 hr. Intracellular DIG was imaged 24 h later by confocal microscopy (Figure 3A). DIG-labeled siRNA (green) was readily detected (estimated >80%) of the HAE. No green signal was detected in the HAE cells treated with non-electroporated exosomes (Figure 3B). These results indicate that exosomes are capable of delivering siRNA into HAE. 

To verify that exosome delivered siRNA can bring about functional knockdown of its target RNA, we chose the ubiquitously expressed transcript hypoxanthine-guanine phosphoribosyl-transferase (HPRT) [42]. We electroporated exosomes derived from A549 cells with either scrambled or HPRT specific siRNA. The level of knockdown was compared to unelectroporated (control) exosomes. As before, exosomes were applied to the basolateral side of HAE for 4 h. HPRT mRNA levels were quantified 24 h later by qRT-PCR. Compared to controls, HPRT mRNA levels were significantly decreased by ~40% in cells that received HPRT-specific siRNA (Figure 3C). These findings indicate that exosomes can successfully deliver siRNA into HAE. 

### 3.4. Protein Delivery to HAE Using Exosomes

**Figure 4:**
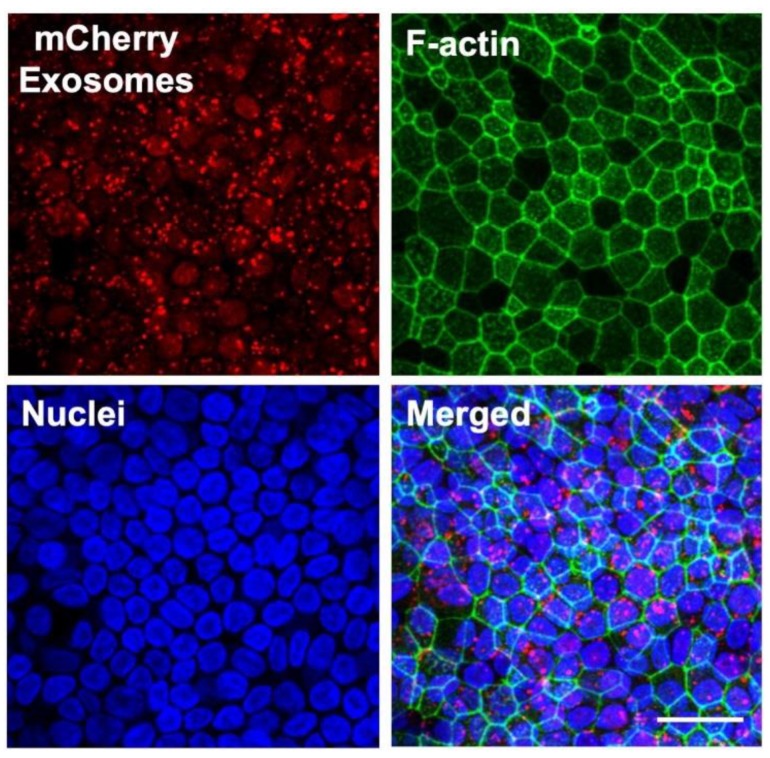
Exosome-mediated protein delivery to well-differentiated HAE. HAE were treated basolaterally for 4 h with exosomes derived from HEK-293T cells that were transfected with a plasmid expressing mCherry protein (red). After 24 h, HAE were fixed, permeabilized, and then stained for F-actin (red) and nuclei (blue). Scale bar = 50 µm.

To evaluate the ability of exosomes to deliver proteins into HAE, we loaded the exosomes with the fluorescent reporter protein mCherry. To do this, HEK-293T cells were transfected with a plasmid expressing mCherry. Three days later, exosomes were collected from the supernatant. HAE were treated basolaterally with purified exosomes for 4 h and the presence of mCherry in HAE was examined 24 h later using confocal microscopy. We readily observed intracellular mCherry signal in treated cells indicating uptake of exosomes by HAE (Figure 4). The mCherry signal remained prominently cytoplasic. Together these data strongly suggest that exosomes are a useful delivery tool in HAE.

### 3.5. MV-Mediated Delivery of CFTR Protein Corrects Anion Defect in HAE from CF Donors

CFTR complementation restores the anion channel defect in well-differentiated human airway epithelial cells derived from CF donors (HAE^CF^) [43,44]. Typically, viral vectors are required to deliver the *CFTR* cDNA. Here we test whether CFTR protein can be delivered to HAE^CF^ using either exosomes or MVs and functionally correct the Cl^−^ channel defect. For these experiments, A549 cells were first transduced with Ad-vector (MOI = 50) expressing either mCherry or CFTR. MVs or exosomes were then purified from the A549 supernatant one day later as described (Figure 1A).

Thus, far we focused our efforts on using exosomes to deliver siRNA or protein to HAE. Exosomes are typically 80–150 nm in diameter and will readily pass through the 400 nm pores of the Transwell inserts on which HAE are grown. MVs range in size from 200 to 1000 nm. However, exosomes typically package proteins <150 kDa; whereas, MVs can package proteins as large as ~300 kDa. The fully glycosylated, mature form of CFTR is ~250 kDa. Thus, for CFTR delivery, we sought to contrast exosomes and MVs while allowing equivalent access to the basolateral surface. To achieve basolateral access with an apical application, we pretreated HAE with 0.1% lysophosphatidylcholine (LPC). LPC is a natural airway surfactant that transiently opens tight junctions and allows access to the basolateral surface. LPC treatment is a common strategy to improve transduction efficiencies of viral vectors, such as VSV-G pseudotyped lentivirus [45] or adenovirus [44], with preferences for the basolateral surface of polarized epithelia. 

**Figure 5:**
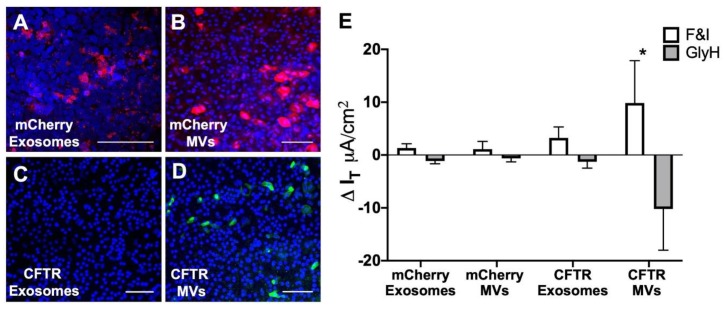
MV-mediated correction of chloride (Cl^−^) transport in cystic fibrosis (CF) human primary epithelia. HAE^CF^ were treated apically with A549-derived EVs: (**A**) mCherry exosomes, (**B**) mCherry MVs, (**C**) CFTR exosomes, or (**D**) CFTR MVs for 4 h. HAE^CF^ were pretreated with 0.1% LPC for 2 h before EVs treatment. After 24 h, HAE^CF^ were fixed, permeabilized, and examined using confocal microscopy for mCherry (red) or immunostained for CFTR protein (green). Nuclei were stained with DAPI (blue). Scale bar = 100 µm. (**E**) Transepithelial Cl^−^ current was measured in HAE^CF^ treated with either mCherry-EVs or CFTR-EVs in Ussing chambers. Change in current was measured in response to forskolin and IBMX (F&I) and GlyH-101. Data are represented as means and s.d. of two HAE cultures from three donors, * *p* < 0.05.

EVs (exosomes or MVs) loaded with mCherry were applied to the apical surface of LPC treated HAE. Intracellular localization of mCherry was examined 24 h later by confocal microscopy (Figure 5A,B). We observed equivalent levels of mCherry expression in HAE treated with MVs or exosomes. We next loaded EVs with CFTR and applied them to the apical surface of LPC treated HAE^CF^. Cells were fixed and immunohistochemisty was used to detect CFTR protein 24 h after EV application. In this case, CFTR protein was only detected following application of MVs but not exosomes (Figure 5C,D). In addition, the bioelectric properties of epithelia were analyzed in Ussing chambers. HAE^CF^ treated with CFTR MVs showed a significant increase in transepithelial Cl^−^ current in response to forskolin and 3-isobutyl-1-methylxanthine (F&I) and current was inhibited by the CFTR channel blocker, GlyH-101 (Figure 5E) when compared to cells treated with either mCherry MVs, mCherry exosomes, or CFTR exosomes. These data demonstrated that MV-mediated apical delivery of CFTR protein to primary airway epithelia can correct the anion channel defect in vitro as early as 24 h post treatment.

## 4. Discussion 

HAE prepared from donor trachea and bronchi are grown at an air–liquid interface and form a pseudostratified columnar epithelium with tight junctions. The cultures include multiple cell types such as ciliated cells, non-ciliated cells, goblet cells, and basal cells that recapitulate the surface cells of the conducting airways [2,46]. These cells provide an excellent model for studying airway cell biology. Indeed, HAE recapitulate many in vivo barriers to gene delivery. In the present study, we investigated the utility of extracellular vesicles (EVs) to deliver different cargos to this model system.

Manipulating genes (either by overexpression or knockdown) is a fundamental strategy for examining gene function. However, the transfection efficiency in well-differentiated HAE is reproducibly below the limit of detection regardless of the transfection or electroporation reagent [7,9,47]. The differentiation process correlates with a highly resistant transfection barrier within 5–6 days after seeding; this prevents the entry of oligonucleotides no matter the formulation of the transfection reagent [7,9,47]. Transfection is only possible when conducted at the time of seeding when the cells are still poorly differentiated [9]. Transfecting siRNA into poorly differentiated airway cells leads to knockdown of target genes; however, this strategy has its limitations because poorly differentiated HAE are simply less representative of the in vivo airways. In the time it takes the epithelia to differentiate (3–4 weeks), the effect of the siRNA gene knockdown will diminish. The established technique for achieving efficient gene transfer in well-differentiated HAE is to use viral vectors such as adenovirus, lentivirus, or AAV. Viral vector can be time consuming and expensive when considering the effort to clone packaging plasmids as well as producing, purifying, and titering the vector. 

Due to their small size (~30–100 nm), exosomes are considered nanoparticles. As a class, nanoparticles are used extensively to deliver small molecules, peptides, proteins, DNAs or siRNAs [48]; however, many manufactured lipid-based nanoparticles have associated withxicity and trigger adaptive and innate immune responses [49,50,51,52]. Exosomes are naturally adapted to transmit molecular messages between cells without invoking immune responses and are considered a safe means to deliver small therapeutic agents such as siRNA and drugs to specific target tissues in a non-cytotoxic manner [53]. Several studies demonstrate that an exosome-based delivery system can deliver its cargo to multiple cell types in vitro and in vivo. Exosomes offer significant advantages over synthetic drug delivery systems including enhanced serum stability, low immunogenicity, and minimal clearance by lung, liver, and spleen [29,53,54,55] and have reproducibly been used in vivo to deliver siRNAs or miRNAs [21,56,57,58]. In addition, exosomes will deliver other therapeutic agents, such as doxorubicin [59], STAT-3 inhibitor JSI-124 (cucurbitacinI) [60], cytosine deaminase (CD) fused with uracil phosphorybosyltransferase (UPRT) [61], curcumin [62], and catalase (exoCAT) [63]. Thus, exosomes are an effective delivery tool for multiple materials into multiple cell types.

Our results suggest that exosomes are readily taken up by the cells in well-differentiated HAE and they can efficiently deliver siRNA. We foresee many applications of this delivery system. For example, siRNAs or proteins that affect the levels of CFTR could be used to screen gene modulators, such as *SIN3A*, *DERL1, ARF4, CDH1,* et al. In future studies we aim to push the levels of knockdown further by expressing lung epithelial specific peptides (GFE-1 and GFE-2) [64] on the surface of exosomes and/or use a pH-sensitive fusogenic peptide [65] for efficient delivery of the cargo into HAE.

MVs are the class of EVs that are largest in size, and which therefore carry large protein cargo like CFTR and laminin proteins [66]. In this study, we showed MV-mediated delivery of CFTR protein to correct the transepithelial Cl^−^ current in well-differentiated HAE from CF donors. We used both MVs and exosomes to deliver the CFTR protein, but only MVs were capable of delivering functional CFTR protein to HAE^CF^. Based on the known size constraints of exosomes, we speculate that the large CFTR protein was more efficiently packaged in MVs. However, the mechanisms of how EVs are differentially produced, released, fused in target cells all may be important for function of a membrane bound channel protein. MVs may be more suitable for delivery of any cell surface channel protein regardless of the size. The mechanism of CFTR incorporation is the subject of future studies and will include western blot and super resolution microscopy analysis. In addition, for our studies we primarily used MVs and exosomes derived from A549 cells. Different EV donor cell lines may lead to different results. 

A limitation of EVs purified from cultured cells is the presence of unintentional cellular protein contamination. In general, MVs are more promiscuous for inclusion of cellular proteins than exosomes. In this study, we did not test for the presence of unintentional proteins or nucleotides from the donor cells. However, EV mediated transfer of DNA fragments are shown to be unstable in target cells [29]. 

We and others have observed that some enveloped viruses, such as measles virus, have a strong preference for basolateral entry into HAE [67]. However, other viruses, such as Rous Sarcoma Virus, preferentially enter the apical surface. Currently it is unclear why EVs have a strong preference for entry at the basolateral surface. We speculate that proteins necessary for EV entry may be basolaterally localized. EVs are likely to play an important role in cell-to-cell communications in lung biology and disease. The lung is a unique organ with a multitude of regionally segregated epithelial cell types; as well as, endothelial and resident immune cells. Exosomes derived from airway cells play a role in innate defense and remodeling [68,69]; however, other cells (such as resident macrophages and neutrophils) are also a significant source of exosomes in the lung. Exchange of EV cargos between these cells can alter gene expression and may reorganize the airway epithelium. It is currently unclear why we observed a clear entry preference for EVs at the basolateral surface of HAE; however, this may represent a bona fide region of interface. 

In summary, our study showed the potential use of EVs in manipulating the gene expression in an important in vitro airway model system. Exosomes effectively deliver siRNAs to modulate endogenous gene expression, whereas MVs can be used to deliver large protein cargos. There are many potential applications of this technology in HAE, including: screening of siRNAs and miRNAs to modulate gene expression, delivering sgRNA libraries for screening genetic phenotypes, generating models for rare CFTR mutations, and delivering 100–150 bp single-stranded DNA oligonucleotides.

## Figures and Tables

**Table 1:** Sequences of DsiRNA used in the study. DNA bases are in bold and 2 O’ methyl bases are underlined. Amino dT bases coupled to DIG are in italics.

DsiRNA	Target mRNA	DsiRNA Target Sequence
hHPRT	human HPRT	5′ pGCCAGACUUUGUUGGAUUUGAA**TT**
		3′ UCGGUCUGAAACAACCUAAACUUUAA
DIG-HPRT	human HPRT	5′ pCCAGUAAAGUUA*T*CACAUGUUCUA**G**
		3′ GUGGUCAUUUCAAUAGUGUACAAGAUC
Scrambled	Not applicable	5′ pCGUUAAUCGCGUAUAAUACGCGU**AT**
		3′ CAGCAAUUAGCGCAUAUUAUGCCGCAUA
